# *Fusarium graminearum FgCWM1* Encodes a Cell Wall Mannoprotein Conferring Sensitivity to Salicylic Acid and Virulence to Wheat

**DOI:** 10.3390/toxins11110628

**Published:** 2019-10-29

**Authors:** Ya-Zhou Zhang, Qing Chen, Cai-Hong Liu, Lu Lei, Yang Li, Kan Zhao, Mei-Qiao Wei, Zhen-Ru Guo, Yan Wang, Bin-Jie Xu, Yun-Feng Jiang, Li Kong, Yan-Lin Liu, Xiu-Jin Lan, Qian-Tao Jiang, Jian Ma, Ji-Rui Wang, Guo-Yue Chen, Yu-Ming Wei, You-Liang Zheng, Peng-Fei Qi

**Affiliations:** 1 State Key Laboratory of Crop Gene Exploration and Utilization in Southwest China Sichuan Agricultural University, Chengdu 611130, Sichuan, China; zhangyazhou@stu.sicau.edu.cn (Y.-Z.Z.); qiantaojiang@sicau.edu.cn (Q.-T.J.); jirui.wang@gmail.com (J.-R.W.); guoyuech74@hotmail.com (G.-Y.C.); ylzheng@sicau.edu.cn (Y.-L.Z.); 2 Triticeae Research Institute, Sichuan Agricultural University, Chengdu 611130, Sichuan, China; qingchen83@sicau.edu.cn (Q.C.); rainbow@stu.sicau.edu.cn (C.-H.L.); leilu@stu.sicau.edu.cn (L.L.); liyang1@stu.sicau.edu.cn (Y.L.); zhaokan6137@163.com (K.Z.); 2018212010@stu.sicau.edu.cn (M.-Q.W.); guozhenru@stu.sicau.edu.cn (Z.-R.G.); wangyan6@stu.sicau.edu.cn (Y.W.); binjiexu@outlook.com (B.-J.X.); jiangyunfeng2018@163.com (Y.-F.J.); kongli@sicau.edu.cn (L.K.); liuyanlin@sicau.edu.cn (Y.-L.L.); lanxiujin@163.com (X.-J.L.); jianma@sicau.edu.cn (J.M.)

**Keywords:** *Fusarium* head blight, defense, mannose, mycotoxin, pathogen

## Abstract

*Fusarium graminearum* causes *Fusarium* head blight (FHB), a devastating disease of wheat. Salicylic acid (SA) is involved in the resistance of wheat to *F. graminearum*. Cell wall mannoprotein (CWM) is known to trigger defense responses in plants, but its role in the pathogenicity of *F. graminearum* remains unclear. Here, we characterized *FgCWM1* (*FG05_11315*), encoding a CWM in *F. graminearum*. *FgCWM1* was highly expressed in wheat spikes by 24 h after initial inoculation and was upregulated by SA. Disruption of *FgCWM1* (Δ*FgCWM1*) reduced mannose and protein accumulation in the fungal cell wall, especially under SA treatment, and resulted in defective fungal cell walls, leading to increased fungal sensitivity to SA. The positive role of *FgCWM1* in mannose and protein accumulation was confirmed by its expression in *Saccharomyces cerevisiae*. Compared with wild type (WT), Δ*FgCWM1* exhibited reduced pathogenicity toward wheat, but it produced the same amount of deoxynivalenol both in culture and in spikes. Complementation of Δ*FgCWM1* with *FgCWM1* restored the WT phenotype. Localization analyses revealed that FgCWM1 was distributed on the cell wall, consistent with its structural role. Thus, *FgCWM1* encodes a CWM protein that plays an important role in the cell wall integrity and pathogenicity of *F. graminearum*.

## 1. Introduction

Common wheat (*Triticum aestivum* L.) is one of the most important cereal crops worldwide. *Fusarium* head blight (FHB) leads to dramatic yield losses and mycotoxin (deoxynivalenol, DON) contamination of wheat seeds, which threatens human and animal health [1,2]. The filamentous fungus *Fusarium graminearum* is the main causal agent of FHB in wheat [3]. Despite the major economic and health impacts of FHB, there is no efficient strategy to manage this disease, partly because we have not understood the biology of *F. graminearum* well [4,5].

**Figure 1:**
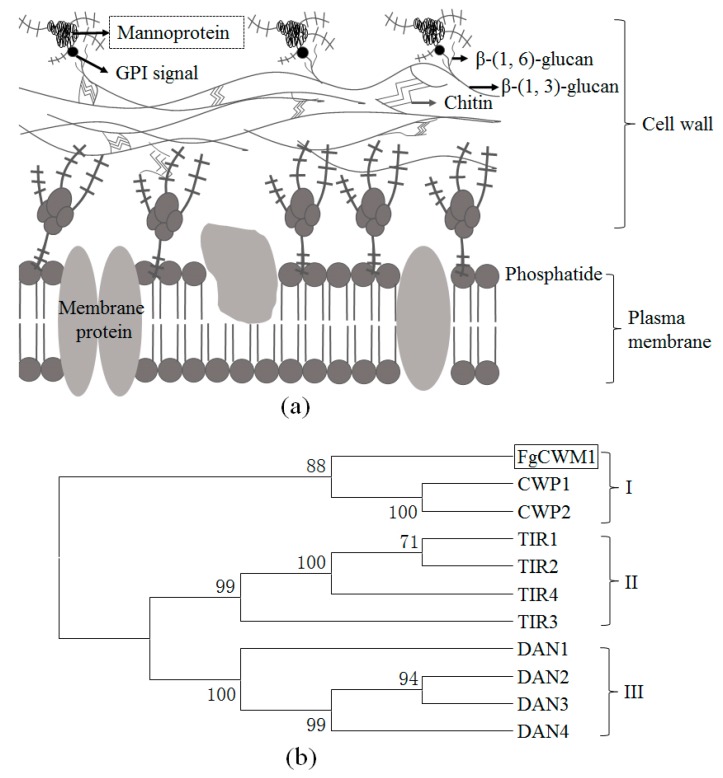
(**a**) Model of fungal cell wall with a layered structure [6,9,10]. The inner, electron-transparent layer is mainly composed of chitin, β glucan (β-(1,3)-glucan and β-(1,6)-glucan). The β-(1,3)-glucan is in contact with chitin, and β-(1,6)-glucan is in contact with glycosylphosphatidyl inositol (GPI) residues. The outer, electron-dense layer is composed of cell wall mannoproteins (CWMs) that are in contact with GPI. (**b**) Neighbor-joining tree of FgCWM1 protein and nine CWMs in *Saccharomyces cerevisiae* [11,15,16]. Genbank accession numbers for CWP1, CWP2, TIR1, TIR2, TIR3, TIR4, DAN1, DAN2, DAN3, and DAN4 are NP_012827, NP_012826, KZV11760, AJU12981, KZV10607, KZV07893, NP_012684, EGA81792, EGA56320, and KZV10416, respectively.

The cell wall is an important component of fungi. It is not only involved in biofilm formation, cell wall biogenesis, and protection against environmental factors, but is also the first point of contact with the host. Thus, it plays an important role in fungi/host interactions [6,7,8]. Fungal cell walls have a layered structure [6,9,10] (Figure 1a), including the inner, electron-transparent layer and the outer, electron-dense layer. The outer, electron-dense layer is mainly composed of cell wall mannoproteins (CWMs), and CWMs play important roles in fungal vegetative growth and pathogenicity [11,12,13,14]. In *Saccharomyces cerevisiae*, the knock-out of *CWP1* and *CWP2* genes encoding CWMs increased its sensitivity to abiotic stresses and antimicrobial peptides, and *CWP2* was shown to play a key role in normal cell wall formation [15,16,17]. In *Aspergillus fumigatus*, *AfMnn9* encodes a β-1,6-mannosyltransferase involved in CWM production. Deletion of *AfMnn9* reduced CWM production and cell wall integrity and increased sensitivity to calcofluor white, Congo red, and hygromycin B [18,19]. *Camp65p* encodes a putative CWM adhesin in *Candida albicans*; this protein has a dual role in hyphal cell wall construction and virulence and affects the host’s immune response to *C. albicans* [20]. To date, there are no reports on the role of CWMs in *F. graminearum*.

Salicylic acid (SA) is an important plant hormone that is involved in the defense response of wheat against *F. graminearum* infection [21,22,23,24]. Previous studies have shown that infection of wheat heads with *F. graminearum* results in a significant increase in SA accumulation [25] and that SA signaling is critical for FHB resistance in wheat [26,27]. Moreover, SA treatments have been shown to decrease the germination efficiency and mycelial growth of *F. graminearum* and decrease DON production [22]. In our previous study, we found that SA downregulated the expression of *FgLAI12* (linoleic acid isomerase gene) and *FgCHS8* (chitin synthase gene) in *F. graminearum*; these genes encode components of the fungal cell membrane and cell wall, respectively, and are essential for the fungal response to stress conditions, including SA [28,29]. However, *F. graminearum* can efficiently export and metabolize SA [23,24,30] to reduce its toxicity. The data known are still not enough to explain the molecular mechanisms of *F. graminearum* in response to SA [22].

In this study, we characterized *FgCWM1* (*FG05_11315*), encoding a CWM in *F. graminearum. FgCWM1* expression was found to be upregulated by SA and was strongly induced in wheat heads during *F. graminearum* infection [31]. The objective of this study was to understand the role of this CWM in *F. graminearum* by analyzing the function of *FgCWM1* and to clarify the role of *FgCWM1* in fungal pathogenicity. These findings will be helpful for understanding the mechanism of *F. graminearum* in response to wheat endogenous SA and the role of CWM in the wheat/*F. graminearum* interaction.

## 2. Results

### 2.1. Sequence Analysis

**Figure 2:**
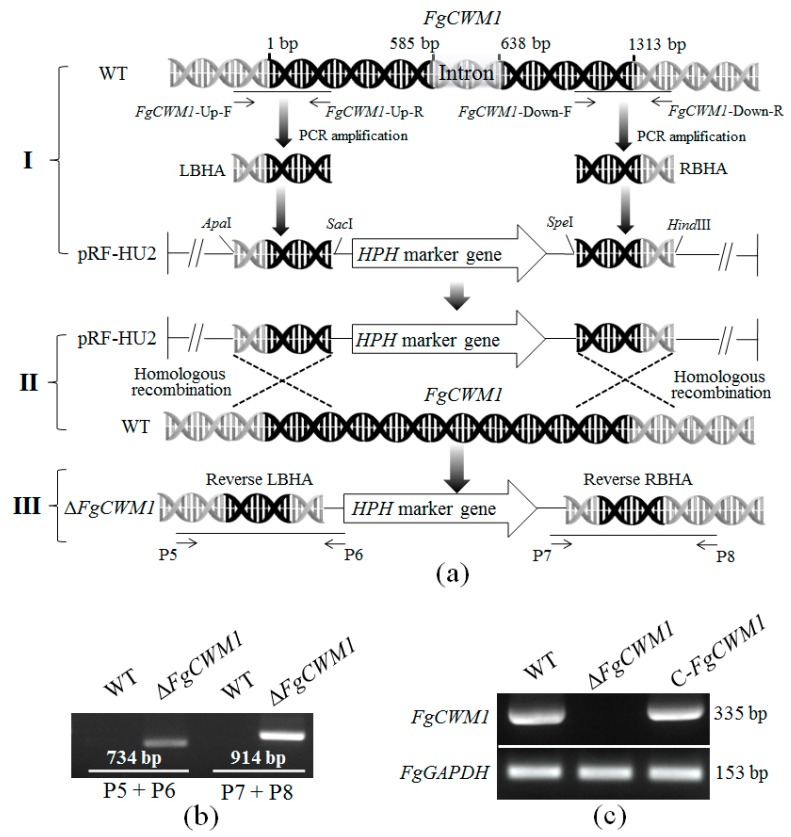
Creation of Δ*FgCWM1* and C-*FgCWM1* mutants. (**a**) Schematic for disruption of *FgCWM1* (Δ*FgCWM1*). *Sac*I, *Apa*I, *Spe*I, and *Hind*III are restriction enzymes used. Black arrow represents targeted location of primers; black lines show amplified sequences. LBHA, left border homologous arm. RBHA, right border homologous arm. (**b**) Verification of Δ*FgCWM1*. Primer pairs P5 + P6 and P7 + P8 were respectively positioned upstream and downstream of inserted T-DNA sequence of Δ*FgCWM1*. (**c**) Reverse transcription PCR (RT-PCR) verification of *FgCWM1* expression using primer pair RJ-*FgCWM1*-F + RJ-*FgCWM1*-R. *FgGAPDH* gene was used as reference. For PCR primers, see Table 1. All PCR products were verified by sequencing (Qingke, Chengdu, China).

Sequence analyses revealed that the *FgCWM1* gene is 1313 bp in length, with two exons and one intron (part I of Figure 2a), and its full open reading frame is 1260 bp long. *FgCWM1* encodes a putative cell wall mannoprotein (CWM), which is supported by a neighbor-joining tree of the deduced amino acid sequences of CWMs from *S. cerevisiae* (Figure 1b). CWMs can be divided into three groups, i.e., groups I, II, and III. *FgCWM1* falls into group I with *CWP1* and *CWP2*, which encode CWMs in *S. cerevisiae* [11,15,16].

### 2.2. Creation of Δ*FgCWM1* and C-FgCWM1

To disrupt the function of *FgCWM1* in *F. graminearum*, the flanking regions (left border homologous arm (LBHA) and right border homologous arm (RBHA)) of the *FgCWM1* gene were amplified from genomic DNA of *F. graminearum*, and then inserted into the pRF-HU2 vector to prepare a disruption plasmid (part I of Figure 2a). The deletion mutants (Δ*FgCWM1*) were created by replacing the entire *FgCWM1* gene with the target selectable marker hygromycin (*HPH*) through homologous recombination (part II of Figure 2a). To ensure that the construct had integrated at the intended homologous site, primer pairs P5 + P6 and P7 + P8 (part III of Figure 2a) were used to detect the construct in Δ*FgCWM1*. The two primer pairs amplified products with the expected size from Δ*FgCWM1* (Figure 2b). Seven Δ*FgCWM1* mutants were generated and were verified by sequencing (data not shown).

To create complementation mutants (C-*FgCWM1*), the open reading frame of *FgCWM1* was introduced into Δ*FgCWM1*. Six C-*FgCWM1* mutants were used. The WT (wild type), Δ*FgCWM1,* and C-*FgCWM1* strains were verified by RT (reverse transcription)-PCR, using the primer pair RJ-*FgCWM1*-F + RJ-*FgCWM1*-R (Table 1). *FgCWM1* was expressed normally in C-*FgCWM1* as in WT but was not expressed at all in Δ*FgCWM1* (Figure 2c). These results demonstrated that *FgCWM1* was correctly removed from the genome of *F. graminearum* and was successfully re-expressed in C-*FgCWM1*.

### 2.3. Effect of FgCWM1 on Mycelial Growth

**Figure 3:**
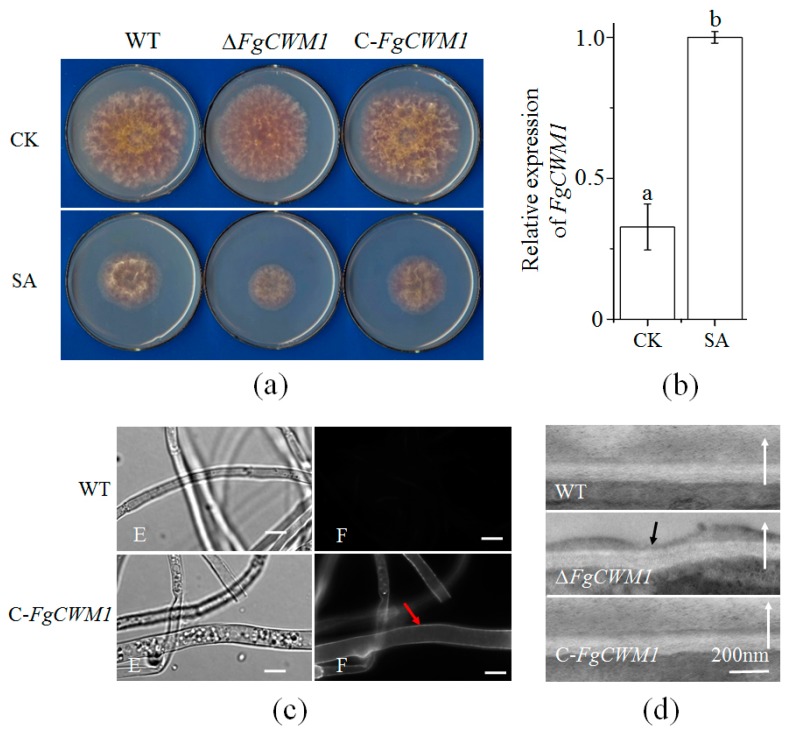
Effect of *FgCWM1* on fungal biology. (**a**) Comparison of mycelial growth on mSNA (modified Synthetischer Nährstoffarmer Agar) plates with salicylic acid (SA, 0.9 mmolL^−^^1^) and without SA (control; CK) on d 5 after inoculation with 1 × 10^3^ conidia (five biological replicates per treatment). (**b**) Relative expression of *FgCWM1* in mycelia under SA and CK treatments in wild-type (WT) strain. Mycelia were collected from plates shown in Figure 3a. Different small letters above columns indicate significant difference (*n* = 5; *p* < 0.05). (**c**) Subcellular localization of FgCWM1 protein, by using the FgCWM1:GFP (green fluorescent protein) fusion protein. E, optical micrograph; F, fluorescence micrograph. Red arrow marks fluorescent protein signal. Scale bar, 10 µm. (**d**) TEM (transmission electron microscope) images of cell walls. White arrows indicate extracellular material. Black arrow marks defective site on cell wall.

To observe the changes in the growth phenotype of *F. graminearum* caused by disruption of *FgCWM1*, mycelial growth of the WT, Δ*FgCWM1,* and C-*FgCWM1* strains was compared on mSNA (modified Synthetischer Nährstoffarmer Agar) plates with or without 0.9 mmolL^−1^ SA (Figure 3a). Δ*FgCWM1* grew slower than WT and C-*FgCWM1* under SA treatment, while their mycelia grew similarly under control conditions (Figure 3a). Consistent with its positive role in response to SA stress, *FgCWM1* expression was induced by SA (Figure 3b). These observations imply that *FgCWM1* participates in the *F. graminearum* response to SA stress.

### 2.4. Effect of FgCWM1 on Fungal Cell Wall Development

The green fluorescent protein (GFP) was tagged to the C-terminal of FgCWM1 in C-*FgCWM1* mutants, which were used to investigate the subcellular localization of FgCWM1 protein. Microscopic observations showed that the FgCWM1 protein is localized on the cell wall (Figure 3c), suggesting that it plays a structural role in the cell wall. To determine the effect of *FgCWM1* on the cell wall, hyphae of WT, Δ*FgCWM1,* and C-*FgCWM1* strains were observed under a transmission electron microscope (TEM). An obvious deficiency in the outer cell wall was observed in the hypha of Δ*FgCWM1*, whereas the cell walls had smooth surfaces in the hyphae of WT and C-*FgCWM1* (Figure 3d).

### 2.5. FgCWM1 Encodes a *Cell Wall Mannoprotein*

**Figure 4:**
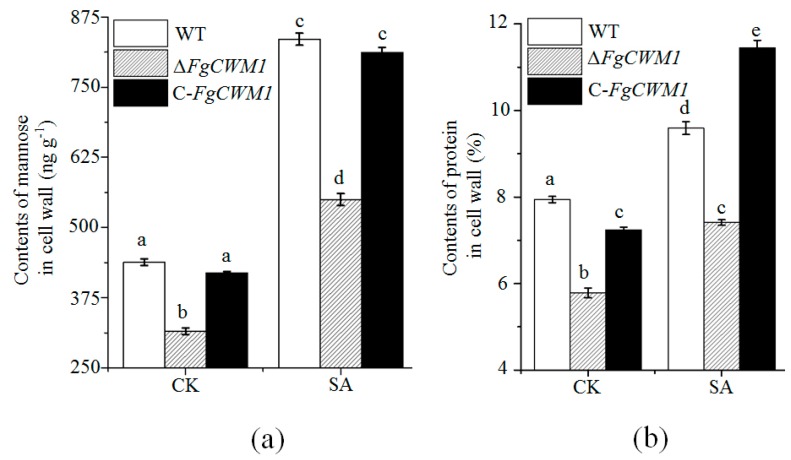
Measurement of mannose and protein contents in cell wall with SA (0.9 mmolL^−1^) and without SA (control; CK). (**a**) Mannose contents in cell wall. (**b**) Protein contents in cell wall. Mycelia were collected from mSNA plates in Figure 3a. Values are average ± standard deviation of three biological replicates per treatment. Different small letters above each box indicate significant difference (*n* = 3; *p* < 0.05).

**Figure 5:**
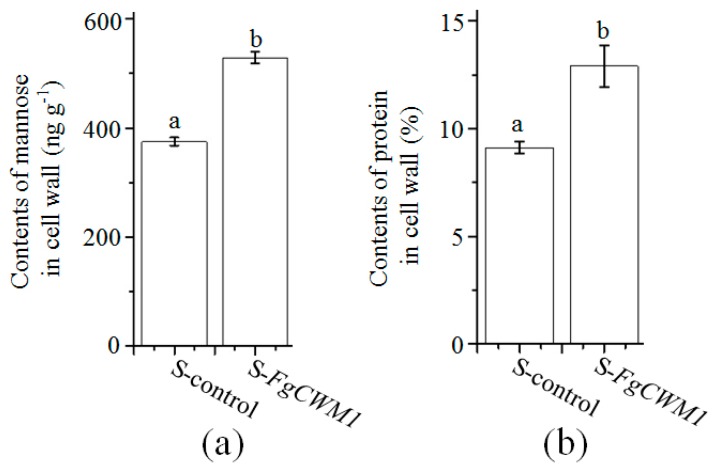
Expression of *FgCWM1* increased accumulation of mannose and protein in *S. cerevisiae*. (**a**) Mannose contents in cell wall. (**b**) Protein contents in cell wall. Values are average ± standard deviation of three biological replicates per treatment. Different small letters above each box indicate significant difference (*n* = 3; *p* < 0.05).

Considering that *FgCWM1* encodes a putative CWM (Figure 1b), the contents of mannose and protein in the cell wall of WT, Δ*FgCWM1,* and C-*FgCWM1* strains were compared. As expected, Δ*FgCWM1* showed significantly lower contents of mannose and protein, as compared with those in WT and C-*FgCWM1* strains (Figure 4a,b). Consistent with the increased expression level of *FgCWM1* under SA treatment, the accumulation of mannose and protein was also increased under SA treatment (Figure 4a,b). To confirm its function, *FgCWM1* was expressed in *S. cerevisiae* (S-*FgCWM1*). The contents of mannose and protein in the P-*FgCWM1* strain were significantly higher than those in the control strain (transformed with empty vector; S-control) (Figure 5a,b).

### 2.6. Effect of FgCWM1 on Pathogenicity

**Figure 6:**
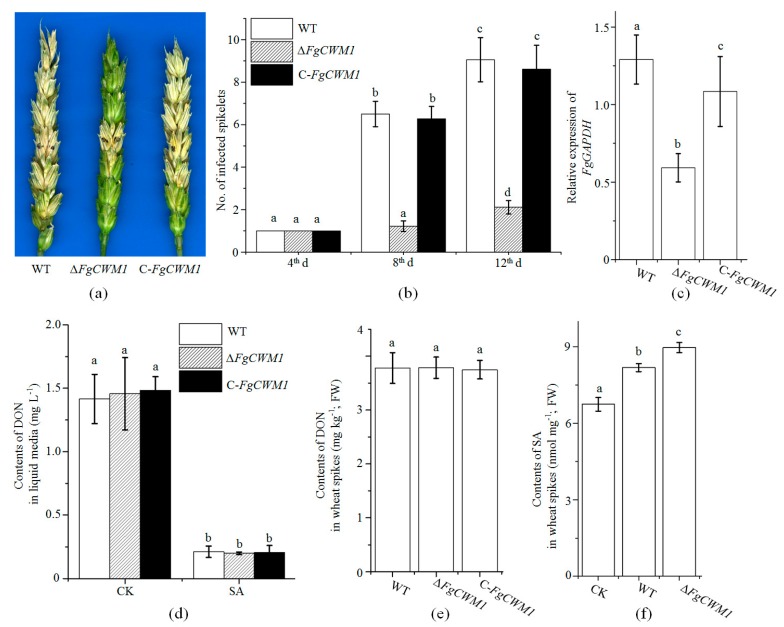
Effect of *FgCWM1* on fungal pathogenicity. (**a**) Wheat heads inoculated with conidial suspensions of wild-type (WT), Δ*FgCWM1*, and C-*FgCWM1* strains. Infected wheat heads were photographed on day 8 after initial inoculation. Black points on spikelets indicate inoculation sites. (**b**) Numbers of infected and bleached spikelets on d 4, 8, and 12 after inoculation. (**c**) Relative expression of *FgGAPDH* in wheat spikes at 24 h after initial inoculation. (**d**) Contents of DON in liquid medium with SA (0.9 mmolL^−1^) and without SA (control; CK). There are three biological replicates for each fungal strain under both CK and SA treatments. (**e**) Contents of DON in wheat ears. (**f**) Levels of SA in spikes inoculated with water (CK), WT, and Δ*FgCWM1* at 24 h after inoculation. FW, fresh weight. Values in (b–f) are average ± standard deviation of three biological replicates per treatment. Different small letters above each box indicate significant difference (*n* = 3; *p* < 0.05).

To determine whether *FgCWM1* participates in the pathogenicity of *F. graminearum*, two fully developed florets of a central spikelet were point-inoculated with conidial suspensions of WT, Δ*FgCWM1,* and C-*FgCWM1*, respectively. Spikes inoculated with Δ*FgCWM1* showed much milder disease symptoms and less fungal biomass, as compared with those inoculated with WT and C-*FgCWM1* (Figure 6a–c). However, the DON contents in the liquid culture medium and in wheat spikes did not differ significantly between Δ*FgCWM1* and WT (Figure 6d,e).

Considering that *FgCWM1* was induced by SA and was highly expressed by as early as 24 h after initial inoculation in wheat heads [31], and that *FgCWM1* was found to influence the fungal response to SA stress (Figure 3a), we compared the SA contents between spikes inoculated with WT and those inoculated with Δ*FgCWM1* (Figure 6e). The spikes inoculated with Δ*FgCWM1* accumulated more SA than did those inoculated with WT.

## 3. Discussion

In fungi, CWM, which is located on the outer cell wall, is an important component of the cell wall (Figure 1a). The CWM is essential for fungal vegetative growth and pathogenicity [11,12,13]. In *F. graminearum*, *FgCWM1,* which encodes a CWM, was found to affect mycelial growth under SA stress (Figure 3a). As expected, localization analyses showed that the FgCWM1 protein is localized on the cell wall (Figure 3c). Consistent with the distribution of CWMs in fungal cell wall (Figure 1a), the absence of *FgCWM1* led to reduced contents of mannose and protein in the cell wall, resulting in a defective outer cell wall (Figure 3d), and consequently, dramatically reduced pathogenicity in wheat (Figure 6a,b). Our results demonstrate that *FgCWM1* encodes a CWM that plays an important role in the host–pathogen interaction between *F. graminearum* and wheat.

There are nine *CWM* genes in the *S. cerevisiae* genome, which are divided into three classes. *FgCWM1* is in group I (Figure 1b). In our unpublished transcriptome data, *FgCWM1* was the only *CWM* gene upregulated by SA. However, as shown in Figure 4, SA was able to significantly induce the accumulation of mannose and protein in the cell wall of *F. graminearum,* even in Δ*FgCWM1*, which lacked the *FgCWM1* that contributes to the accumulation of mannose and protein. This result suggests that some other *CWM* gene(s) are present in the genome of *F. graminearum*.

Fungal cell walls have a layered structure, and CWM and chitin are the two major components [6,9,10] (Figure 1a). Chitin and CWM are distributed in the inner and outer layers of the cell wall, respectively. Reduction of chitin biosynthesis by deleting the gene encoding chitin synthase resulted in a different and almost invisible inner layer in cell walls of *Fusarium asiaticum* [32]. In the present study, deletion of *FgCWM1* resulted in an obvious deficiency in the outer cell wall of *F. graminearum* (Figure 3d). In *F. graminearum,* chitin is synthesized by chitin synthase, including *FgCHS8* (chitin synthase gene) [28]. Considering the importance of CWM and chitin in fungal cell wall integrity, and the toxicity of SA to *F. graminearum* [22], we compared the expression of *FgCHS8* and *FgCWM1* under SA stress. Treatment with SA resulted in downregulation of *FgCHS8* and upregulation of *FgCWM1* [28] (Figure 3b), suggesting that *F. graminearum* can overcome the toxicity of exogenous SA by enhancing the accumulation of CWM in the outer cell wall, even though SA is able to weaken the inner cell wall structure.

*FgCWM1* is a valuable gene target for controlling FHB disease. The recently cloned *Fhb1* gene can lead to a substantial reduction in the severity of visual FHB disease symptoms in wheat spikes [33,34], and *Fhb1* has been widely and successfully used for wheat breeding in China for a long time. In this study, spikes inoculated with Δ*FgCWM1* showed mild and non-spreading FHB disease symptoms and much lower fungal biomass, as compared with those inoculated with WT and C-*FgCWM1* (Figure 6a–c). Host-induced gene silencing (HIGS) is a promising way to inhibit *F. graminearum* infection of wheat [35,36]. Considering its significance in fungal pathogenicity, *FgCWM1* is a promising gene target for enhancing wheat resistance against FHB by HIGS.

In wheat, SA is involved in resistance against FHB [21,23,24,37,38]. This plant defense hormone triggers systemic acquired resistance and induces the expression of a set of defense-related genes [26,27]. The SA content in wheat spikes was found to be significantly increased in response to *F. graminearum* infection [25]. SA directly affects *F. graminearum,* resulting in decreased DON production, lower germination efficiency, and reduced mycelial growth [22]. In this study, Δ*FgCWM1* grew slower than WT and C-*FgCWM1* under SA stress, while SA induced the expression of *FgCWM1* (Figure 3b), suggesting that *FgCWM1* is not a target of SA during the inhibition of mycelial growth. Although the SA content is increased in infected spikes, *F. graminearum* has the ability to export and metabolize SA to avoid SA toxicity [22,23,24,30]. Furthermore, we observed that *F. graminearum* is able to strengthen the outer cell wall by upregulating the expression of *FgCWM1* (Figure 3b) under SA stress. This is despite the SA-induced downregulation of *FgLAI12* (linoleic acid isomerase gene) and *FgCHS8* in *F. graminearum*, which encode important components of the fungal cell membrane and cell wall, respectively, and are essential for the fungal response to SA [28,29]. Compared with spikes inoculated with the WT strain, those inoculated with Δ*FgCWM1* accumulated more SA (Figure 6f), confirming the key role of *FgCWM1* in the fungal response to wheat endogenous SA. The wheat defense response against *F. graminearum* also involves SA signaling [21,26,27]. However, Qi et al. [39] reported that exogenous application of SA at 1 mmolL^−1^ to wheat flowering heads induced only two genes. Therefore, we speculate that the direct effect of SA, rather than SA signaling, plays a more important role in wheat resistance against FHB disease.

Grain contamination by mycotoxin released by *F. graminearum* is a significant threat to the health of animals and humans [1,2]. Because SA significantly decreases the production of DON, it is a promising phytohormone for reducing mycotoxin contamination (Figure 6d). However, the gene controlling DON production that is targeted by SA remains unclear. In FHB, the severity of visual disease symptoms is not strongly related to the amount of mycotoxin in grains. Therefore, resistance to spreading and resistance to toxins are recognized as two different types of resistance in wheat [40]. We found that the expression level of *FgCWM1* was strongly related to the severity of visual FHB symptoms (Figure 6a), but not to DON production (Figure 6d) under these experimental conditions. Therefore, *FgCWM1* is not involved in regulating the biosynthesis of DON in *F. graminearum*.

## 4. Materials and Methods

### 4.1. Materials and Growth Conditions

The *F. graminearum* isolate DAOM180378 (Canadian Fungal Culture Collection, Agriculture and Agri-Food Canada, Ottawa, ON, Canada), which is highly virulent in wheat, was used in all experiments. To produce conidia, the fungus was cultured in carboxymethyl cellulose (CMC) medium at 28 °C, with shaking (180 rpm) for five days [41]. *F. graminearum* was cultured on modified SNA (mSNA; 1 g KH_2_PO_4_, 1 g KNO_3_, 0.5 g MgSO_4_, 0.5 g KCl, 1 g glucose, 1 g sucrose, and 20 g agar per liter) plates at 25 °C. Each plate was inoculated with 1 × 10^3^ conidia of *F. graminearum* and all inoculated plates were incubated in a dark cabinet at 28 °C. A stock solution of SA (1 molL^−1^) was prepared in methanol and added to media after autoclaving. *Agrobacterium tumefaciens* strain AGL-1, used for transforming *F. graminearum*, was grown at 28 °C in yeast extract broth (YEB; 5 g nutrient broth, 1 g yeast extract, 5 g peptone, 5 g sucrose, and 0.2 g MgSO_4_ per liter; pH 7.4). *S. cerevisiae* strain AH109 was grown on yeast peptone dextrose adenine (YPDA) medium (10 g yeast extract, 20 g dific-peptone, 20 g glucose, and 0.03 g adenine sulfate per liter). Unless specifically noted, all chemicals were purchased from Sigma-Aldrich (St. Louis, MO, USA).

Wheat (*Triticum aestivum* cv. ‘Roblin’) plants were grown in a greenhouse under a 16/8 h (day/night) photoperiod at 23/18 °C. Plants were watered as needed and fertilized before planting with 15–15–15 (N–P–K) compound fertilizer. ‘Roblin’ is highly susceptible to *F. graminearum* infection.

### 4.2. Sequence Analysis

The gene sequence of *FgCWM1* (*FG05_11315*) was downloaded from the Ensemble database (http://fungi.ensembl.org/index.html). Primer Premier (version 5.0; Premier Bio soft, Palo Alto, CA, USA) was used to design PCR primers (Table 1). Reported CWM protein sequences were downloaded from NCBI (National Center for Biotechnology Information; http://www.ncbi.nlm.nih.gov). Their deduced amino acid sequences were aligned using MEGA version 5 [42]. Neighbor-joining trees (10,000 replicates) for classification of deduced proteins were constructed using MEGA software, with Poisson correction and complete deletion of gaps (Figure 1b).

### 4.3. Construction of Deletion and Complementation Mutants

Genomic DNA was extracted from mycelia cultured on mSNA plates for 5 d at 28 °C, by the CTAB (Cetyltrimethylammonium bromide) method [43]. The deletion of *FgCWM1* from the genome of *F. graminearum* is illustrated in Figure 2a. The pRF-HU2 vector was used for targeted gene replacement in *F. graminearum* through *A. tumefaciens*-mediated transformation [44,45]. Transformation of *F. graminearum* was carried out as described elsewhere [46]. For complementation, the coding region of *FgCWM1* was ligated into pCAMBIA1302 vector (with the green fluorescent protein gene (m*GFP5*) tag) to make the FgCWM1::mGFP5 fusion construct, which was then transformed into Δ*FgCWM1* (Figure 2b).

### 4.4. *FgCWM1* Expression in S. cerevisiae

To express *FgCWM1* in *S. cerevisiae*, the full open reading frame of *FgCWM1* (without the termination codon; amplified by the primer pair SS-*FgCWM1*-F + SS-*FgCWM1*-R) was inserted into the pYC54 vector, and transformed into *S. cerevisiae* strain AH109 (TIANDZ, Beijing, China) following the manufacturer’s instructions.

### 4.5. Determination of Mannose and Protein Contents in Fungal Cell Wall

Cell walls were prepared and extracted as per Kollár et al. [47] with some modifications. Fresh mycelia of *F. graminearum* in Figure 3a (0.2 g) and cells of *S. cerevisiae* (0.2 g) were washed three times with 0.9% NaCl, and then suspended in 2 mL Tris-HCl buffer (50 mmolL^−1^; pH = 7.5) with 0.5 g glass beads (0.5 mm diameter, Sigma-Aldrich, St. Louis, USA). Mycelia samples were shaken at 30 s^−1^ for 3 min (Retsch MM400, Haan, Germany). Cell wall material was pelleted by centrifugation (1500× *g*) for 10 min and then washed three times with Tris-HCl buffer at room temperature.

Mannose was extracted from the cell wall pellet as described by Cameron et al. [48], with some modifications. The collected cell wall pellet was resuspended in 2 mL 0.1 molL^−1^ citrate buffer solution (pH = 6.6; 0.1 molL^−1^ citric acid and 0.1 molL^−1^ sodium citrate) and incubated in a sterilization pan (Sanyo MLS-3780, Tokyo, Japan) at 121 °C for 3 h. The solution was separated by centrifugation (900 g) for 20 min at 4 °C. The supernatant was transferred into a 10 mL centrifuge tube and mixed with triploid precooled ethanol (with 1% acetic acid) at 4 °C for 12 h. Mannose was obtained by centrifugation (900 g) and then suspended in 200 μL water. The concentration of mannose was assayed using an enzyme-linked immune response kit, following the manufacturer’s instructions (Jin Yibai Biological Technology Company, Nanjing, China). The protein contents in the cell wall were determined as in [49].

### 4.6. Microscopic Assay

For optical microscopic and fluorescence microscopic assays, 1000 conidia of WT and C-*FgCWM1* were respectively inoculated into 3 mL mSNA liquid medium (1 g KH_2_PO_4_, 1 g KNO_3_, 0.5 g MgSO_4_, 0.5 g KCl, 1 g glucose, and 1 g sucrose) and cultured at 28 °C on an orbital shaker at 120 rpm for two days. Mycelia were collected and observed under a Nikon-80i fluorescence microscope (Nikon, Tokyo, Japan) to determine the subcellular localization of FgCWM1.

For transmission electron microscope (TEM) observations, mycelia of WT, *∆FgCWM1,* and C-*FgCWM1* were harvested as above and fixed [50]. Cells were permeated with 812 epoxy resin monomers (SPI-Pon™ 812, West Chester, USA), cut using a Leica UC7 microtome (Leica microsystems, Wetzlar, Germany), and analyzed and photographed using an Hitachi HT7700 TEM (Hitachi, Tokyo, Japan).

### 4.7. Virulence Assay and DON Measurement

To determine the effect of *FgCWM1* on the pathogenicity of *F. graminearum* in wheat heads, two flowering florets of a central spikelet of one head were each inoculated with 1 × 10^3^ conidia. The inoculated heads were wrapped in moist plastic wrap and incubated for 48 h at 25 °C. The FHB symptoms were assessed 2–12 days after inoculation at 25 °C. There are three biological replicates per treatment, and each replicate contains at least five heads.

A two-stage protocol was used to test whether *FgCWM1* is related to the production of DON in liquid media [22,51]. The effect of *FgCWM1* on DON production in wheat heads was determined as described elsewhere [24]. The wheat samples were collected on the sixth day after inoculation. There are three biological replicates (with at least two heads) for each treatment. The amount of DON was measured using a DON ELISA kit (Beacon, ME, USA) and a Multiskan Spectrum instrument (Thermo Fisher Scientific, Waltham, MA, USA).

### 4.8. Gene Expression Analysis

Total RNA was extracted from fresh powdered material (mycelia or wheat spikelets ground in liquid nitrogen) using the E.Z.N.A.^®^ Total RNA Kit I (Omega Bio-Tek, Norcross, GA, USA) according to the manufacturer’s instructions. Then, RNA was reverse transcribed using the PrimeScript™ RT Reagent Kit with genomic DNA Eraser (Takara, Dalian, China) following the manufacturer’s protocol.

The primer pair Rj-*FgCWM1*-F + Rj-*FgCWM1*-R was used to measure the expression level of *FgCWM1* in *F. graminearum*. The glyceraldehyde 3-phosphate dehydrogenase (*FgGAPDH*, *FG05_06257*), β-tubulin (*FG05_09530*), and elongation factor 1 (*FG05_08811*) genes were used as reference genes when performing qPCR for *F. graminearum* samples [22]. The relative amount of *F. graminearum* was estimated by measuring the expression level of *FgGAPDH* in wheat spike tissue by qPCR, with normalization against three wheat reference genes (*w-GAPDH*, NCBI UniGene Ta.66461; *Aox*, Ta.6172; *hn-RNP-Q*, Ta.10105) [22]. The qPCRs were performed using a MyiQ Real-Time PCR Detection System (Bio-Rad, Hercules, CA, USA). All of the primers mentioned above are listed in Table 1.

### 4.9. Quantification of SA in Wheat Spikes

To prepare wheat spike samples, two florets from each fully developed spikelet in a whole spike at the mid-anthesis stage were inoculated with 1 × 10^3^ conidia or water. The inoculated wheat plants were treated as described above. At 24 h after inoculation, the spikes were harvested and ground to a fine powder in liquid nitrogen. Three biological replicates per treatment were analyzed. The quantification of SA was conducted as described by Siciliano et al. [52].

### 4.10. Statistical Analysis

Student’s *t*-test (implemented in DPS (Data Procession System) version 12.01 software (Zhejiang University, Hangzhou, China); [53]) was used to test the significance of differences among average values of cell wall mannose content, cell wall protein content, relative expression levels of genes, DON content, and disease level.

## Figures and Tables

**Table 1:** Primers used in this study.

Primer	Sequence (5′– 3′)	Reference
*FgCWM1*-Up-F	GCGGGCCCTACTCAGGGTAACGGAAAGG	This study
*FgCWM1*-Up-R	GCGAGCTCACAATGCAGCTCAATGTCG	
*FgCWM1*-Down-F	GGAAGCTTCAACCCAGACCTACCCA	This study
*FgCWM1*-Down-R	GGACTAGTGAAGAGCAGCGAACCAG	
P5	TGATAATAATGTCCTCGTTCC	This study
P6	TGACGAACTGTAAGTCGGATA	
P7	ACCGAACTTCAAGACACCA	This study
P8	CAACGGCCTCAACCTACT	
R-*FgCWM1*-F	AACCATGGATGAAGTTCTCCGCTGC	This study
R-*FgCWM1*-R	GGCCATGGAAGCATCCTTCAGAAGAGGT	
SS-*FgCWM1*-F	GCGAGCTCGCATGAAGTTCTCCGCTGC	This study
SS-*FgCWM1*-R	GGGGATCCAAGCATCCTTCAGAAGAGGT	
Fg-*GAPDH*-F	TGACTTGACTGTTCGCCTCGAGAA	[22]
Fg-*GAPDH*-R	ATGGAGGAGTTGGTGTTGCCGTTA	
Fg-*β-tubulin*-F	GTTGATCTCCAAGATCCGTG	[22]
Fg-*β-tubulin*-R	CATGCAAATGTCGTAGAGGG	
Fg-*Factor1*-F	CCTCCAGGATGTCTACAAGA	[22]
Fg-*Factor1*-R	CTCAACGGACTTGACTTCAG	
RJ-*FgCWM1*-F	GCTGGTGCCGAGGCTGTT	This study
RJ-*FgCWM1*-R	GGTCATCGCAGGTGTTTCCA	
*Aox*-F	GACTTGTCATGGTAGATGCCTG	[22]
*Aox*-R	CAGGACGAGCATAACCATTCTC	
w-*GAPDH*-F	AACTGTTCATGCCATCACTGCCAC	[22]
w-*GAPDH*-R	AGGACATACCAGTGAGCTTGCCAT	
*hn-RNP-Q*-F	TCACCTTCGCCAAGCTCAGAACTA	[22]
*hn-RNP-Q*-R	AGTTGAACTTGCCCGAAACATGCC	

Restriction enzyme cut sites are underlined.
